# Digital smile revolution: Converging art, science, and technology

**DOI:** 10.6026/973206300213447

**Published:** 2025-10-31

**Authors:** Denisse Carreras, Yogalakshmi Devendrababu, Vidhya Balasubramanian, Shruti Bassi, Simarpreet Sandhu, Bhavesh Kumar Sharma

**Affiliations:** 1 Department of Dentistry, Institute Serafin Ruiz De Zarate Ruiz, Santa Clara, Villa Clara, Cuba; 2 Department of Dental Sciences, Sri Ramachandra Institute of Higher Education and Research, Tamil Nadu, India; 3 Department of Dentistry, Adhiparasakthi Dental College and Hospital, Melmaruvathur, Tamil Nadu, India; 4 Department of Dentistry, Punjab Government Dental College and Hospital Amritsar, Punjab, India; 5 Department of Dental Sciences & Research, Maharishi Markandeshwar College of Dental Sciences and Research, Haryana, India; 6 Department of Prosthodontics, Chaudhary Charan Singh University, Uttar Pradesh, India

**Keywords:** Digital smile design (DSD), aesthetic rehabilitation, aesthetic smile redesign, multidisciplinary rehabilitation

## Abstract

Smile design integrates scientific and artistic principles to craft aesthetically pleasing smiles based on patient data, dental research, and individual perceptions of beauty influenced by cultural or ethnic factors. Esthetics has become a key expectation in dental treatments, reflecting the increasing demand for attractive smiles. A smile has a significant impact on social interactions, influencing emotional expression and societal function. Digital Smile Design (DSD) is an advanced, interdisciplinary planning tool that enhances diagnostic clarity, patient communication and treatment predictability. By systematically analyzing facial and dental characteristics, DSD enables comprehensive, personalized and predictable outcomes in esthetic dentistry.

## Background:

Dental aesthetics constitutes an essential component of contemporary dentistry, influencing far more than just the visual improvement of one's smile. It involves a multifaceted relationship between factors affecting psychological health, social interactions, functional capability and overall quality of life. Recognizing its broad significance underscores the need for comprehensive dental care that effectively integrates aesthetic goals with functional and health-related considerations [1]. Psychological well-being is closely associated with self-esteem and confidence. Individuals who feel satisfied with their dental appearance tend to have greater self-esteem, enabling them to participate more confidently in both social and professional situations [2]. Facial appearance significantly influences social interactions and psychological well-being, shaping human personality. Among facial features, the eyes and mouth are most frequently linked to attractiveness [3]. Everyone desires a confident and attractive smile. Digital Smile Design (DSD) is a tool that clinicians use when patients aspire to achieve their ideal smile but feel uncertain and struggle to visualize the treatment outcome. The primary goal of Digital Smile Design is to assist clinicians by providing an improved aesthetic perspective that directly addresses patient concerns, educating and encouraging them regarding the advantages of the proposed treatment. By simulating and previewing the anticipated results, the digital platform creates a visual representation of the final smile. This approach actively involves patients in their smile transformation, allowing customization according to their personal preferences and requirements. Consequently, this personalized approach aligns closely with the psychological profile of each patient, enhancing their confidence in the treatment process and increasing their acceptance of the planned dental procedures. Digital Smile Design (DSD) is an innovative, flexible dental planning technique developed by Brazilian dentist Christian Coachman in 2007. It enables dental professionals to digitally craft a patient's smile using a series of photographs taken before and after the DSD process [4]. Additionally, the DSD software serves as an educational tool, demonstrating potential aesthetic enhancements to patients and facilitating the collection of their personal preferences and expectations. This interactive approach encourages patients to actively participate in the decision-making process, ensuring they feel involved rather than passive recipients of dental care [5]. More than a technique, DSD represents a comprehensive philosophy that fosters interdisciplinary collaboration and emotional engagement through tools like smart adaptive technologies and intelligent data [6]. Therefore, it is of interest to report and describe its clinical applications, digital workflow, and overall contribution to contemporary aesthetic dentistry.

## Evolution of digital smile designing:

Over the past two decades, smile designing has rapidly evolved from traditional physical analogue methods to advanced digital technologies, progressing from 2D to 3D designs. Initially, clinicians relied on hand-drawn illustrations on printed patient photographs to demonstrate potential treatment outcomes. Today, this approach has fully transitioned to digital drawing, utilizing Digital Smile Design (DSD) software, which enables clinicians to effortlessly edit or revise designs, ensuring the outcome meets both aesthetic and functional patient expectations [7]. The Evolution in generations was proposed by Christian Coachman in 2017 as follows [8]:

[1] Generation 1: Analogue drawings over the images, and there was no connection to the analogue model. This was the period when drawing was carried out on a printed copy of photographs to picture the treatment result, but there was no correlation with the study model.

[2] Generation 2: Two-dimensional digital drawings and visual connection to the analogue model. With the arrival of the digital world, digital drawing was permitted on certain software like PowerPoint. It was more accurate and less time-consuming compared to hand drawing. The diagram could be visually connected to the study model, but there was still a lack of physical connection.

[3] Generation 3: Digital two-dimensional drawings and an analogue connection to the model. This was the commencement of the digital-analog connection. Introduction of the very first drawing software that linked 2D digital smile design to 3D wax-up.

[4] Generation 4: Two-dimensional digital drawings and connection to the 3D model. There was a transition in digital dentistry from 2D to 3D analysis. 3D digital wax up could be carried out involving facial integration and pre-planned dental aesthetics framework.

[5] Generation 5: A Complete 3D workflow was established

[6] Generation 6: The 4D concept was introduced.

## Principles of smile design:

Creating an ideal smile using digital software demands a comprehensive understanding of the muscles involved and the dimensions of the smile display zone, along with their aesthetic proportions. As every individual is unique, each case must be carefully assessed and personalized to achieve the most suitable smile design. These elements are interdependent, and any modification in one aspect will inevitably influence the others. While software algorithms assist in predicting an ideal smile, clinical smile design necessitates a multidisciplinary approach. The aesthetic challenges represented by previous multidisciplinary rehabilitations would not have been successful without perfect communication between the various members of the therapeutic team: prosthetic practitioner, implantologist and orthodontist and laboratory prosthetist [9]. Facial symmetry, facial profile, and proportion of the facial structures are key facial features in planning for esthetic smile redesign. According to the literature, ideal facial proportions suggest that the distance between the two superciliary arches should be equal to the total facial width measured between the zygomatic prominences. Additionally, the intercanthal or pupillary line should align perpendicularly to the Frankfurt horizontal occlusal plane. Vertically, the face is typically divided into three equal segments by drawing imaginary lines: from the glabella to the superciliary arch, from the arch to the nasal tip, and from the subnasale to the menton (chin point) [10]. An ideal smile is built upon the foundation of well-proportioned lips. During a natural smile, approximately 2 mm of the maxillary incisors, along with the interdental papilla, should be visible. Excessive tooth and gum exposure leads to a gummy smile, whereas insufficient exposure can cause the upper lip to appear flattened and create a frowning expression [11].

## Major components and workflow of DSD:

Digital imaging - Digital Smile Design (DSD) begins with capturing high-resolution digital records, including facial photographs, intraoral scans, and radiographs. These serve as the essential baseline for comprehensive analysis and precise treatment planning. Smile Design - With the aid of specialized software, dentists can digitally manipulate images to simulate various smile designs and enhancements. This process involves evaluating the patient's facial and dental proportions and making precise adjustments to create a harmonious and aesthetically appealing smile [4]. Mock-up and wax-up - After finalizing the ideal smile design; a physical mock-up or wax-up is fabricated to provide the patient with a tangible preview of the planned changes. This enables the patient to visualize the expected outcome and provide feedback before any clinical procedures are initiated [12]. Communication and collaboration - DSD enhances communication between the dentist, dental laboratory and interdisciplinary team members. By allowing seamless sharing of digital files, it ensures precise information transfer and promotes effective collaboration in complex cases involving orthodontics, prosthodontics, or periodontics [13]. Patient presentation - DSD enables dentists to present the proposed treatment plan to patients through digital visuals and mock-ups. This visual approach helps patients better understand the anticipated results, align their expectations realistically, and actively engage in the decision-making process [14].

[1] Step 1: Intake and photo session.

[2] Step 2: Digital smile design

[3] Step 3: Wax up

[4] Step 4: Try in

[5] Step 5: Treatment plan.

[6] Step 6: Aesthetic procedures

[7] Step 7: Photo session, ideal smile [15]

## Benefits of digital smile design:

DSD enables both dentists and patients to preview the anticipated outcome before beginning any treatment. This visual insight helps align the patient's expectations with the proposed plan, hence informed decision-making regarding their dental care 16]. DSD helps dentists to communicate effectively their treatment plans to patients, hence fostering trust, reducing anxiety and ensuring a shared understanding of the desired outcome [17]. It empowers dentists to enhance precision and predictability in treatment planning. Through digital image manipulation, they can refine the design, explore multiple options and optimize both the aesthetic and functional outcomes of the final restoration [18]. Digital Smile Design promotes effective collaboration among various dental specialties involved in comprehensive treatment planning. The seamless sharing of digital files and streamlined communication enable efficient coordination, leading to improved treatment outcomes [15]. DSD greatly enhances patient satisfaction by actively involving them in the treatment planning process. By valuing and incorporating their input and preferences, patients feel more empowered and confident in their decisions regarding care [19].

## Limitations of digital smile design:

Effective implementation of DSD requires dental practices to invest in essential technologies, including intraoral scanners, digital imaging systems, and licensed software. These investments can be expensive and demand regular maintenance and periodic updates [20]. While DSD serves as a powerful visualization tool, the outcome may not always precisely replicate the digital design. Variations in dental materials, individual patient anatomy, and software limitations can influence the accuracy of the results [21]. Implementing DSD demands that dental professionals develop new technical competencies and become adept at operating the related software and equipment. The learning curve can differ from one practitioner to another and often necessitates dedicated training and hands-on experience [18].

## Conclusion:

Digital Smile Design is revolutionizing aesthetic dental care by integrating advanced technology with interdisciplinary collaboration to offer personalized and predictable treatment outcomes. By enhancing diagnostic precision and facilitating clear communication, DSD fosters greater patient engagement and informed decision-making. While challenges related to data integration and professional training persist, the growing influence of artificial intelligence continues to shape the future of aesthetic dentistry, making it more dynamic and patient-focused than ever before.
